# Hybrid machine learning forecasting for resilient and sustainable pharmaceutical supply chains under regulatory and seasonal disruption

**DOI:** 10.3389/frai.2026.1803863

**Published:** 2026-05-20

**Authors:** Khalid Yahya, Mehdi Safaei, Saleh Al Dawsari

**Affiliations:** 1 Department of Electrical and Electronics Engineering, Faculty of Engineering and Architecture, Istanbul Gelisim University, Istanbul, Türkiye; 2 Faculty of Economics, Administrative, and Social Sciences, Logistics Management Department, Istanbul Gelisim University, Istanbul, Türkiye; 3 School of Engineering, Cardiff University, Cardiff, United Kingdom; 4 Electrical Engineering Department, College of Engineering, Najran University, Najran, Saudi Arabia

**Keywords:** demand forecasting, explainable AI, hybrid model, pharmaceutical supply chain, residual learning

## Abstract

**Introduction:**

Demand forecasting in pharmaceutical supply chains is not a simple task. In regulated markets it becomes more difficult, because seasonality, epidemic waves, and also policy changes can make demand behavior unstable. This study proposes a hybrid residual learning approach for forecasting pharmaceutical demand in Türkiye.

**Methods:**

The model uses Support Vector Regression (SVR) together with Deep Neural Networks (DNN). In this structure, SVR estimates the main or baseline part of demand, and DNN tries to learn the remaining nonlinear residual variation. The framework was tested with real ERP-based demand data, including 10 critical pharmaceutical products in a 24-month period. For evaluation, temporal holdout testing was used. The model was also compared with ARIMA, Prophet, Random Forest, XGBoost, and LSTM. Statistical validation was done by paired t-tests and Diebold–Mariano tests.

**Results:**

The proposed SVR–DNN framework performed better than the standalone SVR and DNN models. It also stayed as the strongest model when it was compared with the wider benchmark models. The hybrid model gave the lowest forecasting errors, and it showed the highest explanatory performance among the tested models.

**Discussion:**

The results show that when baseline demand estimation is separated from residual correction, forecasting performance can be improved in pharmaceutical demand conditions that are sensitive to disruptions. SHAP analysis also helped the interpretation of model, because it showed the main factors that affect demand variation. The proposed framework gives an interpretable and context-aware forecasting model for pharmaceutical supply chains working under regulatory and seasonal disruption.

## 1 Introduction

The pharmaceutical area is an important part of the healthcare system. In this area, supply chain performance is related with several outcomes in operation. Medicine availability is one of them. Inventory over time is another one. The reliability of drug distribution is also affected by this performance. These points are connected to each other and they depend on how the supply chain works in practice (Gorani, 2024). In pharmaceutical operations, demand forecasting still remains necessary. Demand can vary at a high level. Price controls also have an effect. Reimbursement arrangements do as well. Other administrative barriers are also involved, and they affect the flow and supply of pharmaceutical products (Mills and Kanavos, 2023; Seyhun, 2025). Demand forecasting therefore remains necessary in pharmaceutical operations. This is because several objectives need to be handled together at the same time. Service continuity must be kept. Inventory must be controlled in an efficient way. Regulatory conditions also need to be followed. When forecasting is weak, or not available, keeping this balance in actual operations becomes harder (Douaioui et al., 2024; Gorani, 2024).

Recent market data further highlight the growing importance of accurate forecasting in the Turkish pharmaceutical sector. In 2024, Türkiye’s pharmaceutical market was reported at approximately USD 11 billion, with nearly 2.6 billion units sold through retail and hospital channels (Erdağ and Yeğenoğlu, 2025; Gorani, 2024). In value terms, the market grew by 53.8% compared with the previous year, while total sales volume declined by 3.9% (Gorani, 2024). This divergence between value growth and volume contraction reflects the increasing complexity of pharmaceutical planning in Türkiye. Pricing adjustments, reimbursement structures, and regulatory constraints interact with demand variability in ways that may reduce the effectiveness of conventional forecasting approaches (Tengiz et al., 2023). More recent analyses published in 2025 continue to describe the Turkish pharmaceutical market as a tightly regulated access environment shaped by approval procedures, pricing rules, reimbursement decisions, and administrative barriers (Çalışkan, 2025; Seyhun, 2025).

Traditional forecasting methods like ARIMA and exponential smoothing usually depend on linearity and stationarity assumptions, but in pharmaceutical demand this situation often does not stay valid (Carbonneau et al., 2008; Fourkiotis and Tsadiras, 2024). In this kind of market, demand is not always stable and also not fully regular over time. On the other hand, machine learning methods such as SVR and DNN are more able to catch nonlinear relations, find some hidden patterns, and respond better when demand changes in an irregular way (Douaioui et al., 2024; Rathipriya et al., 2023). These models can perform better especially when the pattern does not continue in the same form. But even with this strong potential, many ML-based studies still examine sales history in a narrow way and they do not include important external factors which have serious effect on medicine consumption, and this is still a common weakness in many existing works (Zhu et al., 2021; Douaioui et al., 2024).

In Türkiye, pharmaceutical supply chain is regulated under a mixed public and private regulatory regime. The Ministry of Health, through its centralized administrative system, keeps the control on medicine prices, reimbursement systems, and also procurement procedures (Tengiz et al., 2023). This model has three elements, and these create difficulties for accurate forecasting, because they make fixed pricing boundaries, they cause local market demand imbalances, and also they introduce administrative procedures which take long time (Mills and Kanavos, 2023; Seyhun, 2025). The environment is very dynamic, but at the same time it is also highly regulated, so forecasting systems are needed which are not only data based, but also flexible in practice and explainable as well (Douaioui et al., 2024).

The research gap considered in this study has three sides. First, many usual forecasting approaches, including univariate statistical models like ARIMA and other statistical methods, are limited in their ability for capturing nonlinear demand disturbances coming from epidemic waves, holidays, and regulatory interventions (Fourkiotis and Tsadiras, 2024; Ramadhan et al., 2026). Second, machine learning and deep learning models, such as SVR, DNN, LSTM, XGBoost, and also broader ensemble approaches, have shown promising results, but most earlier studies still rely on standalone models or weak hybrid forms, and they do not use clearly a residual learning structure which can separate baseline demand from irregular context driven fluctuations (Ramadhan et al., 2026). Third, only limited evidence is available from highly regulated emerging pharmaceutical markets like Türkiye, where centralized pricing, reimbursement controls, and procurement rigidities make forecasting conditions that are significantly different from those in more open retail environments (Seyhun, 2025). Based on this, the present study contributes to this issue by developing a context aware hybrid SVR-DNN framework with residual learning, and this framework is evaluated on real ERP-based pharmaceutical demand data from Türkiye.

The main novelty of the proposed framework is its sequential residual learning structure. This is different from usual hybrid models that combine forecasting parts at the same time or depend on fixed decomposition forms. In the proposed SVR and DNN model, the first step is to estimate the main or dominant demand pattern. After that, the remaining nonlinear changes are passed to the DNN part. So the second model does not try to learn everything from the beginning. This step by step structure gives a more specific role to the second learner. It focuses on the residual movements and the remaining volatility, especially the fluctuations related to local disruptions such as epidemic periods, policy changes, and holiday effects. In other words, it works on what is left after the baseline pattern is already estimated. This makes the learning task narrower, and in some sense more focused also. From this view, the proposed model is not the same as hybrid methods like ARIMA and LSTM or other ensemble models based on decomposition (Giannopoulos et al., 2025). The difference is that the present framework gives more importance to targeted residual correction in a pharmaceutical forecasting setting where context matters a lot. It is not only hybrid in a general sense. It uses the second stage for the remaining unstable part of demand, and this is where the model shows its main difference.

From a scientific perspective, the advantage of this framework is not limited to combining two forecasting models. Its main value lies in assigning distinct learning roles to the two components. SVR is used to estimate the dominant and relatively stable baseline demand structure, while DNN is used only for the remaining nonlinear residual variation. This separation is important in disruption-sensitive pharmaceutical forecasting because it allows the second-stage learner to focus on irregular context-driven demand shifts rather than relearning the full demand process from the beginning. In this sense, the proposed framework contributes not only through forecast improvement, but also through a more structured and interpretable treatment of pharmaceutical demand dynamics.

Following this rationale, this paper proposes a hybrid forecasting approach with a two-stage structure, using actual ERP system-based pharmaceutical demand data from a national pharmaceutical distributor in Türkiye. The hybrid approach was assessed with respect to a variety of product groups in a real-world setting. The originality of this research can be identified on three levels: first, a hybrid approach with a residual structure that suits disruption-driven pharmaceutical demand forecasting was proposed; second, external contextual factors were incorporated, as they are usually neglected in forecasting research; third, SHAP values were incorporated to increase the usability of the forecasting approach.

## 2 Literature review

### 2.1 Theoretical background and research gap

The pharmaceutical industry has depended on traditional statistical models which include ARIMA and exponential smoothing and classical linear regression for demand forecasting during multiple years. The existing methods in literature have strong foundations but they depend on three main conditions which include linear relationships and stable data patterns and residual distributions that follow a normal distribution pattern. The pharmaceutical industry faces challenges with these assumptions because disease outbreaks and regulatory changes and seasonal health patterns create rapid changes in customer demand (Gorani, 2024; Schisa and Farnè, 2025).

Recent studies focus more on machine learning algorithms, because these algorithms can learn non-linear time patterns and they adapt when structure of demand changes. The SVR model can handle high dimensional inputs and it remains robust against outlier data, mainly because of its *ε* insensitive loss function. Deep neural networks DNN improve forecasting power by learning complex hidden interactions and also long term dependencies inside time series data, which in many cases helps prediction (Fourkiotis and Tsadiras, 2024; Rezki and Mansouri, 2023).

Previous literature suffers from at least three major drawbacks. The first drawback is that single models like SVR or DNN are utilized, while no hybrid model approach is ever considered. Secondly, many studies use artificial datasets or datasets that have time-limited information. Finally, relevant external factors that directly affect drug demand are ignored despite their influence on drug demand (Gorani, 2024; Douaioui et al., 2024), such as epidemic warning signals, the effect of holidays, or macro-events.

To address these problems there is a three-fold research gap that this study seeks to address for effective time series forecasting. Firstly, current statistical methods are not designed to handle time series that have disruptive phenomena, i.e., sharp and non-linear shifts caused by various external factors such as epidemic waves, holidays and regulatory interventions. This problem is particularly pronounced in pharmaceutical demand time series that are very sensitive to product disruption (Fourkiotis and Tsadiras, 2024). Second, while existing approaches for the second problem type have employed regression models including Support Vector Regression (SVR), Deep Neural Networks (DNN) and LSTMs as well as ensemble methods like XGBoost, they are typically developed as independent models. In contrast, here we explore the potential of incorporating residual learning in order to better distinguish between baseline demand and anomalies (Ramadhan et al., 2026). Third, empirical evidence for highly regulated emerging markets, such as those observed within the Pharmaceuticals Market in Türkiye, is currently lacking despite the pronounced effects of central pricing and reimbursement decisions and procurement practices on market demand determinants (Seyhun, 2025; Tengiz et al., 2023). The existing models have several limitations and deficiencies. To address these shortcomings, in this paper, we propose a context aware hybrid SVR-DNN framework with residual learning approach using real pharmaceutical demand data from Türkiye.

### 2.2 Comparative empirical studies

Despite the recent advances achieved in this field, demand forecasting is one of the strategic pillars of the supply chain management. Mistakes in demand forecasting have great potential to cause huge financial losses. In the pharmaceutical market, such mistakes may even bring serious health effects to the patients. To continue patients’ therapy on time and to avoid expiring unused medicines, the accurate demand forecasting is essential for increasing efficiency and for complying with the relevant legislation (Gorani, 2024; Schisa and Farnè, 2025).

The time series forecasting methods, such as moving averages, exponential smoothing and ARIMA, are the most commonly used techniques in the pharmaceutical setting, as they are traditional and well-established in the literature. They are generally deterministic and are not designed to handle non-linear relationships, sudden shifts in demand or strong seasonality in medicine demand. In recent years, researchers have started to investigate the use of machine learning techniques to improve the accuracy of medicine demand forecasting, in order to obtain a more data-driven approach for handling real-world scenarios (Fourkiotis and Tsadiras, 2024; Mbonyinshuti et al., 2024).

Table 1 summarizes representative studies in pharmaceutical and related supply chain demand forecasting and highlights not only the models used, but also the dominant learning structures adopted in prior work.

**Table 1:** Comparative studies in pharmaceutical and supply chain demand forecasting and learning structures.

Author(s) and year	Dataset and context	Model(s) used	Learning structure	Key result
(Mbonyinshuti et al., 2024)	Public health supply chain	ARIMA vs. LSTM	Standalone comparison	LSTM outperformed ARIMA in capturing complex trends
(Aburto and Weber, 2007)	Chilean retail chain	Hybrid ANN + ARIMA	Parallel hybrid	Hybrid model improved forecast accuracy
(Mirshekari et al., 2024)	Single-pharmacy drug sales data	Bayesian Kernel GPR	Standalone	Bayesian GPR significantly outperformed ARIMA and ANN
(Carbonneau et al., 2008)	Synthetic supply chain demand	SVR	Standalone	SVR managed noise better than traditional regression
(Zhu et al., 2021)	Pharmaceutical drug sales time series	ARIMA + LSTM	Combined hybrid	Hybrid ARIMA–LSTM outperformed standalone models
(Schisa and Farnè, 2025)	Weekly demand for respiratory drugs	MBB-RF, LSTM, Prophet	Standalone comparison	LSTM and MBB-RF excel in capturing non-linear fluctuations
(Fourkiotis and Tsadiras, 2024)	SKU-level pharmaceutical demand	XGBoost vs. ARIMA/LSTM	Benchmarking	XGBoost excelled in seasonality and volatility management
(Ramadhan et al., 2026)	Systematic review (pharma demand)	ML and DL review	Meta-analysis	ML techniques are promising for non-linear characteristics
(Giannopoulos et al., 2025)	Intermittent demand in supply chain	ML algorithms review	Systematic review	ML models show superiority in irregular demand patterns

As shown in Table 1, prior pharmaceutical demand forecasting studies have explored both standalone models and hybrid combinations, including ANN–ARIMA, ARIMA–LSTM, and RF–ARIMAX type structures. However, these studies are predominantly based on standalone learning, parallel hybridization, or decomposition-type combinations. Explicitly residual-based sequential hybrid architectures remain comparatively less represented in this literature, particularly those that first estimate the baseline demand structure and then apply a second learner specifically to the remaining forecast error. It is this more specific methodological gap that the present study addresses through a sequential SVR–DNN residual learning framework.

Deep learning models including LSTM and GRU and CNN LSTM have shown better results for understanding both sequential patterns and market demand patterns. These models successfully monitor long-term time-dependent connections but they require full data availability and their operational accuracy depends on correct parameter settings to stop model overfitting when deployed in actual systems. XGBoost and Random Forest models maintain stability, which makes them popular, but they need feature engineering to properly model seasonality and long-run autocorrelation (Douaioui et al., 2024; Fourkiotis and Tsadiras, 2024; Ramadhan et al., 2026).

To the best of our knowledge, the combined use of SVR and DNN in a residual learning framework has not been sufficiently explored for pharmaceutical forecasting in Türkiye. The Turkish pharmaceutical market operates under specific challenges because of centralized price control, regional disease patterns, and unpredictable procurement schedules. In this context, the proposed hybrid approach seeks to improve operational forecasting by combining SVR-based baseline pattern learning, DNN-based nonlinear residual correction, and contextual information integration (Seyhun, 2025; Tengiz et al., 2023; Çalışkan, 2025).

### 2.3 Recent advances in hybrid models

Hybrid ML models have gained further consideration over the past few years by combining the interpretability of traditional statistical methods with the advanced pattern-recognition capabilities of modern ML algorithms. Hybrid architectures in supply chain forecasting have demonstrated increased robustness, generalizability, and prediction accuracy in a wide range of domains and data sets. Rezki and Mansouri (2023)presented a novel contribution as they proposed a hybrid deep model that combines Recurrent Neural Networks (RNN), Artificial Neural Networks (ANN), and Gradient Boosting to enable risk-aware forecasting in uncertain supply chains. Jahin et al. (2025) also proposed a CNN-GRU-LSTM ensemble model with the capability of learning multi-dimensional time series with in-built interpretability based on SHAP-based analysis. Mulwa et al. (2024) expanded on such ideas through the use of XGBoost with SHAP to forecast healthcare demand influenced by climate in Kenya, both predictive and interpretive in an African supply chain context.

To place the proposed SVR–DNN framework into context against the state of the art, Table 2 provides a comparative overview of selected high-impact papers from 2020 to 2025. These studies highlight the effectiveness of various hybrid architectures in supply chain and pharmaceutical forecasting, with emphasis on both technical performance and integration aspects such as explainability.

**Table 2:** Comparative overview of recent hybrid forecasting architectures (2020–2025).

Author(s) and year	Domain/context	Hybrid model	Key features/dataset	Key insights
(Fourkiotis and Tsadiras, 2024)	Drug sales data (~600 K records)	XGBoost vs. ARIMA + LSTM	ATC clusters for seasonality	XGBoost yielded MAPE ~16–18%, outperforming ARIMA/LSTM
(Rezki and Mansouri, 2023)	Pharma supply chain risk	RNN + ANN + GB	Simulated and real retail datasets	Risk factors improved prediction accuracy and resilience
(Jahin et al., 2025)	General supply chain	CNN + GRU + LSTM	Multi-channel sales with SHAP	SHAP offered actionable interpretability and transparency
(Lin et al., 2025)	Emergency drug demand (earthquakes)	XGBoost + AFT-LSTM	Regional casualty and seasonal variables	Reduced errors vs. Transformer; captured dynamics of casualties
(Ha et al., 2025)	Time series demand (pharma and SC)	STL + LSTM + DNN ensemble	Weekly demand decomposition	Decomposition improved temporal feature extraction accuracy
(El Bilali et al., 2023)	Pan evaporation (interpretability)	DNN + SVR + XGBoost	Hourly climate data	SHAP and LIME identified dominant environmental variables
(Kaushik et al., 2020)	Healthcare expenditures	Ensemble of ARIMA + LSTM + MLP	Weekly expenditure (USD/patient)	Ensemble outperformed standalone models in stability
(Mulwa et al., 2024)	Epidemiological forecasting	XGBoost + SHAP	Healthcare procurement logistics	High accuracy with SHAP-based transparency for decisions
(Khan et al., 2024)	Renewable energy forecasting	LSTM, GRU, CNN-LSTM	Meteorological and temporal features	Optimized LSTM yielded lowest error vs. hybrid combinations
This study	Turkish pharmaceutical demand	SVR + DNN	ERP data and contextual variables	Sequential architecture captures baseline and irregular shocks

As summarized in Table 2, recent hybrid forecasting studies have explored a wide range of integration strategies, including ARIMA–LSTM, decomposition-based LSTM–DNN ensembles, feature-selection plus sequence-learning combinations, and multi-model architectures involving XGBoost, GRU, MLP, or ANN. However, these hybrid structures are predominantly based on ensemble integration, decomposition pipelines, or sequence-intensive designs. In contrast, the present study adopts a residual-based sequential hybrid strategy in which the first learner estimates the dominant baseline demand structure and the second learner is trained specifically on the remaining forecast error. This distinction is important because the contribution of the proposed model lies not merely in combining two learners, but in how the learning roles are separated across baseline extraction and residual correction.

### 2.4 Positioning the hybrid SVR–DNN model

The selection of the SVR–DNN combination was guided by both methodological and data-related considerations. First, the available pharmaceutical demand dataset in this study is relatively short in time horizon and therefore requires a first-stage learner that can capture the dominant demand structure without relying on deep sequence modeling. SVR was considered appropriate for this role because of its robustness under limited-sample nonlinear regression settings.

Second, the residual component of pharmaceutical demand in the Turkish market is shaped by irregular fluctuations associated with holidays, epidemic effects, and policy-sensitive disruptions (Seyhun, 2025; Tengiz et al., 2023). A DNN was therefore used in the second stage to learn the remaining nonlinear correction term after baseline extraction. Compared with alternatives such as SVR–LSTM, the proposed architecture avoids introducing a more sequence-intensive residual learner under limited data conditions. Compared with ARIMA–DNN, it also avoids relying on a strictly linear first-stage baseline when the underlying demand structure may already contain nonlinear variation.

For these reasons, the SVR–DNN design was considered the most appropriate hybrid structure for the present forecasting setting. This model selection also provides a clearer research framework for the present study, because it explicitly links the methodological role of SVR to baseline demand estimation and the methodological role of DNN to residual shock correction. Accordingly, the proposed framework should be interpreted as a structured residual prediction system rather than a generic combination of two forecasting tools. This choice is consistent with the comparative evidence summarized in Table 2, where prior hybrid studies are mainly based on ensemble integration, decomposition pipelines, or sequence-intensive model combinations rather than sequential residual correction logic.

The following section presents the methodological implementation of this sequential residual learning framework and its evaluation strategy.

## 3 Methodology

**Figure 1:**
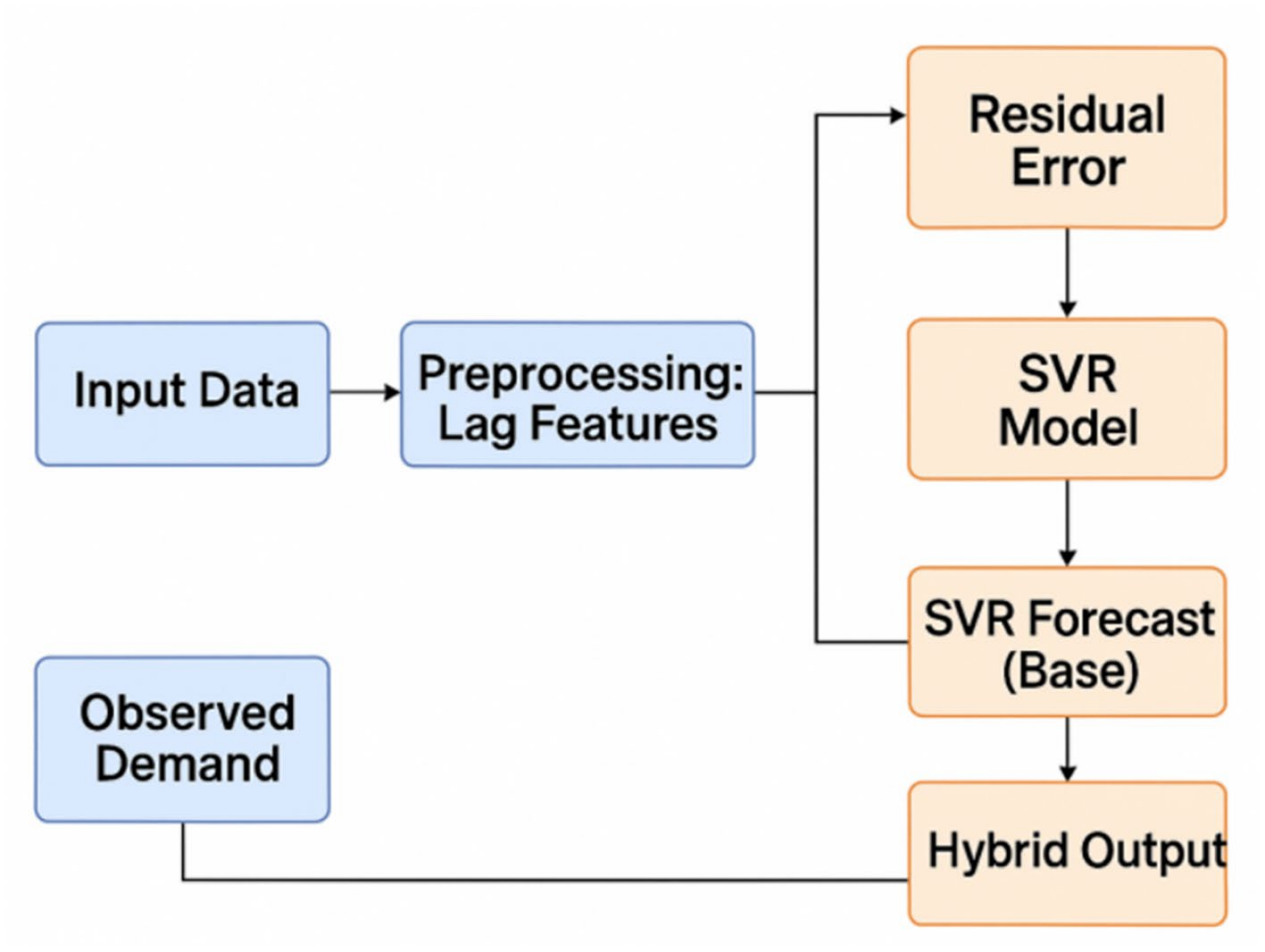
Hybrid forecasting framework (SVR + DNN residual correction).

This section explains data sources, preprocessing pipeline, and machine learning model architecture that are used in this study, including Support Vector Regression SVR, Deep Neural Networks DNN, and proposed hybrid residual learning SVR DNN model. It also presents a simplified visual framework, shown in Figure 1, which explains the modeling procedure step by step and shows how the process is followed in this study.

### 3.1 Study data

In this study, historical demand data from one of the major pharmaceutical distribution companies, which operates all over Türkiye, were used. In the study, monthly historical demand data of 10 high-turnover products that have 24 months of historical data (from January 2022 to December 2023) were used. The products such as antibiotics, painkiller, antihistamine, and cold or flu medicine which are frequently used by patients and have clinical importance and seasonality were selected.

Each observation in dataset contains the product stock keeping unit SKU, the related month, and the quantity ordered by downstream partners such as pharmacies, hospitals, and regional warehouses. The dataset shows some clear demand characteristics. Seasonality is high, especially in winter months for medicines connected to flu. Order quantities are not consistent and smooth because of centralized bulk procurement. Short term demand increases happen suddenly, often because of promotional campaigns or public health emergency cases. There are also time based and regional increases which are connected to disease outbreaks or changes in weather conditions.

A complete preprocessing process was applied to make data valid and also reliable. The data removal step eliminated observations that had three or more consecutive months with missing values or zero values, to remove discontinued SKU items and also possible reporting errors. After this filtering step, 240 valid raw observations remained, corresponding to 10 SKUs observed over 24 months. These observations formed a balanced monthly panel at the preprocessing stage. For supervised forecasting, lagged demand features from *t* – 1 to *t* – 3 were then constructed within each SKU series. As a result, the first three months of each SKU were used only to initialize the lag structure and were not included as forecastable target instances. The final supervised learning dataset therefore included 210 usable observations, coming from 10 SKUs across 21 months, and these observations were then used for model development, validation, and testing.

### 3.2 Data preprocessing

The model training process started with a sequential data preprocessing pipeline, which was designed to keep data stability and also improve interpretability, while accuracy was increased at same time. This pipeline included specific steps for feature normalization, noise reduction in data, and preservation of time series consistency for modeling purpose.

#### 3.2.1 Outlier detection and smoothing

To understand unusual demand increases, outlier detection and smoothing was performed to determine if spikes were a result of inventory shortage, panic buying or caused by a simple data entry error. The IQR method (Fourkiotis and Tsadiras, 2024) was used to determine if the outlier value is an extreme value in a distribution. A rolling median filter with a window size of 3 was also applied to the data in order to smooth the data while preserving important temporal characteristics. After applying the rolling median filter, about 4.2% of the total data points were affected but overall, good preservation of the demand signal’s continuity was achieved.

#### 3.2.2 Handling missing and zero values

After removing products with more than 3 months of zero sales from the dataset, the analysis team further purged the data by removing products/categories where sales data was no longer collected. They also chose to keep in the data the short term zero values as the pattern of zero values were similar to the lows observed in the sales history for that category/product.

#### 3.2.3 Feature scaling

To normalize the input space and to enhance the convergence of the model, Min Max normalization technique is employed to normalize all numerical fields on the train set to values in the range 0 to 1 (Krupakar and Kandappan, 2025). The transformation parameters were saved during preprocessing step, so inverse normalization of predictions could be performed later in evaluation stage, and the results could remain interpretable in original units.

#### 3.2.4 Temporal split

To maintain temporal causality and to repeatedly pose a real-world forecasting problem, the dataset was split in time order forward. Therefore, 80% of the data from the start of the time series in January 2022 until the data from July 2023 were used for training and the remaining 20% of the data from August 2023 to the end of the time series in December 2023 were used for testing. This approach to forward splitting the data prevents future information from being incorporated into the training set, thus avoiding integrity problems and artificially introducing smoothness.

To maintain realism and reduce the likelihood of data leakage, the time series was split 80/20 in time-order strictly. The model was trained on data from January 2022 to July 2023 and evaluated on a fully out-of-sample five-month test horizon from August to December 2023. To further reduce overfitting risk given the limited sample size, hyperparameter tuning was conducted exclusively within the training portion using time-aware cross-validation (Douaioui et al., 2024; Fourkiotis and Tsadiras, 2024). This design ensured that model selection, parameter tuning, and residual construction never accessed future information from the final test set. The five-month test horizon remained intact after lag construction because the lagged predictors for August–December 2023 were computed from demand values observed in the preceding months, all of which were available at the respective forecast origins and did not require any future information.

#### 3.2.5 Feature engineering

Lagged demand features with one-to-three-month delays, from t minus one to t minus three, were constructed to capture auto-correlation dependencies in demand. Because these lagged predictors require prior demand history, the first three observations of each SKU could not serve as forecast targets in the supervised learning matrix. Consequently, although 240 raw monthly observations were retained after filtering, the effective sample size used for model estimation and evaluation was 210 observations after lag construction. Along with the lagged demand variables, three contextual predictors were also included. A calendar month variable $M_{t}$ was used to represent month-of-year seasonality in scalar form. In addition, binary indicators were defined for national public holidays $\left(H_{t}\right)$ and for peak flu season $\left(F_{t}\right)$ , where the flu season covered November to February.

This feature enhancement step created a time series consistent supervised learning data set with internal demand lag features along with external factors. The final modeling matrix had 210 records post lag creation. All preprocessing transformations, including lag generation, Min Max normalization, and temporal splitting, were applied in same fixed and consistent way across SVR, DNN, and hybrid models.

### 3.3 Machine learning model design

This section presents architecture, mathematical formulation, and implementation process of machine learning models used for pharmaceutical demand forecasting, namely Support Vector Regression SVR, Deep Neural Networks DNN, and hybrid SVR DNN ensemble model. All the models were trained using the cleaned and preprocessed data discussed in Section 3.2.

#### 3.3.1 Support vector regression (SVR)

Support Vector Regression (SVR) is a supervised learning technique that predicts the target variable by locating a nonlinear function within an *ε*-insensitive margin. It operates in a high-dimensional feature space and tries to locate a function whose deviations from the actual target values are not more than *ε*, and which is as flat as possible.

The SVR can be expressed mathematically as:

$$f(x)=w^{T}\varphi (x)+b$$ (1)

where: φ(x) : nonlinear mapping to a higher-dimensional feature space; $w^{T}$ : weight vector; b : bias term.

The optimization problem is to minimize the model complexity and penalize the prediction errors outside the *ε*-insensitive tube:

$$\min\frac{1}{2}{\left\|w\right\|}^{2}+C\sum_{i=1}^{n}(\varepsilon_{i}+\varepsilon_{i}^{*})$$ (2)

Subject to:

$$\left\{ \begin{array}{c} y_{i}-w^{T}\varphi (x_{i})-b\leq \varepsilon_{i}+\varepsilon_{i}^{*}\\ w^{T}\varphi (x_{i})+b-y_{i}\leq \varepsilon_{i}+\varepsilon_{i}^{*}\\ \varepsilon_{i},\varepsilon_{i}^{*}\geq 0\; \end{array}\right.$$ (3)

where *C* is the regularization parameter that controls the trade-off between flatness and tolerance to deviations, and $\varepsilon_{i}$ and $\varepsilon_{i}^{*}$ are slack variables for positive and negative prediction errors, respectively.

Nonlinear mapping was performed using the Radial Basis Function (RBF) kernel:

$$K(x_{i},x_{j})=\exp(-\gamma {\left\|x_{i}-x_{j}\right\|}^{2})$$ (4)

SVR hyperparameters were tuned using a grid search algorithm combined with 10-fold time-aware cross-validation. The SVR function is defined in Equation 1, the optimization objective in Equation 2, the constraints in Equation 3, and the RBF kernel in Equation 4.

The search ranges used for tuning are detailed below (Table 3):

**Table 3:** SVR hyperparameter grid.

Parameter	Values tested
*C*	{1, 10, 100}
*ε*	{0.05, 0.1, 0.2}
*γ*	{0.01, 0.1, 1.0}

The systematic tuning process ensured that the SVR model worked at its best under different data arrangements and different lag depth levels. The final SVR configuration selected through grid search was *C* = 10, *ε* = 0.1, and *γ* = 0.01.

#### 3.3.2 Deep neural network (DNN)

The DNN model used in this research has a fully connected feedforward structure, prepared for one step ahead supervised forecasting by using lagged demand values and contextual predictors. The input vector includes past demand observations together with auxiliary contextual features, and the output gives one step ahead forecast for monthly pharmaceutical demand.

Let:

$$X_{t}=[d_{t-1},d_{t-2},d_{t-3},M_{t},H_{t},F_{t}]$$ (5)

where: $d_{t-k}$ : demand at lag *k*; $M_{t}$ : calendar month index; $H_{t}$ : holiday indicator; $F_{t}$ : flu season indicator.

##### 3.3.2.1 Layer structure

The network architecture is built with three layers:

Input layer has 6 neurons, and these represent lag features together with contextual variables.

Hidden layer 1: 64 neurons with ReLU activation.

Hidden layer 2: 32 neurons with ReLU activation.

Output layer: 1 neuron with linear activation for continuous value prediction.

The input structure is defined in Equation 5, while the forward propagation steps are given in Equations 6–8. The loss function is presented in Equation 9. The forward propagation through the network is defined as:

$$z^{\left(1\right)}=\text{ReLU}(W^{\left(1\right)}X+b^{\left(1\right)})$$ (6)

$$z^{\left(2\right)}=\text{ReLU}(W^{\left(2\right)}z^{\left(1\right)}+b^{\left(2\right)})$$ (7)

$$\hat{y}=W^{\left(3\right)}z^{\left(2\right)}+b^{\left(3\right)}$$ (8)

##### 3.3.2.2 Loss function

The model was trained by minimizing mean squared error loss function MSE:

$$\mathrm{MSE}=L=\frac{1}{n}\sum_{i=1}^{n}{\left(y_{i}-{\hat{y}}_{i}\right)}^{2}$$ (9)

where $y_{i}$ is real demand value and ${\hat{y}}_{i}$ is predicted value from model. Optimization was done using Adam optimizer with learning rate set to 0.001. In total 100 training epochs were run, and early stopping was applied after 10 validation epochs without improvement, to avoid overfitting problem. Dropout regularization with rate of 0.2 was applied on hidden layers to improve generalization.

The DNN was implemented using Python 3.9 and TensorFlow–Keras framework. Hyperparameter tuning was carried out through random search over network depth, number of neurons, batch size, and dropout rate, and performance was evaluated using 5 fold time series aware cross validation (see Table 4).

**Table 4:** DNN model architecture.

Layer	Units	Activation	Notes
Input layer	6	—	Lags + temporal/external inputs
Hidden layer 1	64	ReLU	Fully connected
Hidden layer 2	32	ReLU	Fully connected
Output layer	1	Linear	One-step-ahead forecast

##### 3.3.2.3 Training configuration

- Optimizer: Adam
- Learning rate: 0.001
- Loss function: MSE
- Epochs: 100
- Batch size: 16
- Early stopping: patience = 10
- Dropout rate: 0.2

The final DNN configuration used in this study consisted of an input layer with six predictors, two fully connected hidden layers with 64 and 32 neurons, respectively, ReLU activation in the hidden layers, and a linear output layer for one-step-ahead demand prediction. The network was trained using the Adam optimizer with a learning rate of 0.001 for up to 100 epochs. To reduce overfitting, dropout regularization with a rate of 0.2 and early stopping with a patience of 10 epochs were applied. The final batch size selected during random search was 16.

Given the relatively small sample size, several precautions were taken to limit overfitting in the DNN component. The network architecture was intentionally kept shallow, with two hidden layers of moderate size. Dropout regularization (rate = 0.2), early stopping (patience = 10), and a moderate batch size of 16 were used to improve generalization stability. In addition, hyperparameter tuning was restricted to the training data through time-aware cross-validation, while final model performance was assessed on a fully held-out future test period. These design choices were made to reduce memorization of noise, while still keeping enough flexibility for modeling residual volatility.

#### 3.3.3 Hybrid model: SVR + DNN residual learning

The proposed hybrid model uses Support Vector Regression SVR to predict the main demand trend, and then a Deep Neural Network DNN is used to model nonlinear residual parts in a sequential residual learning structure. This model follows a layered approach, so it can capture both stable linear components and also irregular fluctuations, which are common in pharmaceutical demand under contextual disruptions.

The first step in the forecasting methodology is to establish a base demand forecast, using an SVR model to predict the natural demand trajectory over time:

$${\hat{y}}_{i}^{\mathrm{SVR}}=f_{\mathrm{SVR}}(x_{t})$$ (10)

The residual errors, which show unexplained variance, are then calculated in following way:

$$r_{t}=y_{t}-{\hat{y}}_{t}^{\mathrm{SVR}}$$ (11)

After that, a DNN is trained on these residuals by using the same feature set $X_{t}$ , so nonlinear corrections can be learned.

$${\hat{r}}_{t}=f_{\mathrm{DNN}}(X_{t})$$ (12)

The final hybrid prediction is obtained by summing both components together:

$${\hat{y}}_{t}^{\text{Hybrid}}={\hat{y}}_{t}^{\mathrm{SVR}}+{\hat{r}}_{t}$$ (13)

The baseline SVR forecast, the residual definition, the residual correction, and the final hybrid forecast are presented in Equations 10–13, respectively. This two-stage hybrid model combines SVR accuracy in trend forecasting with nonlinear approximation ability of DNN, to produce forecasting results with high performance, especially under situations with sudden demand changes. The hybrid model works very well in real world scenarios where base demand is relatively stable, but external factors such as health initiatives or policy changes introduce sudden fluctuations.

To improve reproducibility, the implementation sequence of the hybrid framework is reported explicitly. First, the SVR model was trained on the original demand series and contextual predictors to estimate the baseline demand component. Second, residuals were computed as the difference between observed demand and SVR predictions. Third, the DNN model was trained on these residual patterns using the same lagged and contextual inputs in order to learn the nonlinear correction term. The final hybrid forecast was obtained by summing the SVR baseline prediction and the DNN residual prediction. All experiments were implemented in Python 3.9 using scikit-learn for the SVR component and TensorFlow/Keras for the DNN component.

During model development, the two-stage hybrid procedure was implemented in a temporally consistent manner. Within each training fold, the SVR component was first fitted using only the observations available in that fold. Residuals were then computed exclusively from the corresponding in-fold SVR predictions. The DNN component was subsequently trained on these fold-specific residuals using the same fold-restricted lagged and contextual predictors. After hyperparameter selection, the final hybrid model was re-fitted on the full training portion, and the final test forecasts were generated only for the held-out future horizon. This procedure ensured that the second-stage learner did not receive information from outside the active training window.

First it makes prediction with SVR, then it learns the residual part using a DNN model, and finally it outputs the corrected final prediction.

#### 3.3.4 Model explainability: SHAP integration

To make deep neural network DNN and hybrid SVR DNN models more transparent and also operationally reliable, they were post processed using SHAP, which stands for SHapley Additive Explanations. SHAP is a consistent game theory based framework that assigns contribution of each input feature to model output in a fair way, and it allows both global and local interpretation of results. This is especially important in regulated industries such as healthcare and pharmaceutical sector, where model reasoning must in our analysis, SHAP values were calculated for all observations in test dataset by using Kernel Explainer method, which is suitable for model agnostic interpretability. Kernel SHAP was chosen because it gives a model agnostic explanation framework that can be applied in a consistent way to both the standalone DNN model and also the complete SVR DNN hybrid pipeline. Gradient based explainers, such as DeepExplainer or GradientExplainer, are more efficient from computational side for pure neural network architectures, but they are less directly suitable for a two stage forecasting system where final output is produced by combination of heterogeneous learners. In this study, interpretive consistency across model components was given more importance than computational efficiency, especially because dataset size was relatively small and there was need to explain final hybrid prediction inside one unified framework. The SHAP analysis supports two levels of interpretation:

- Global insights: finding which features—i.e., lagged demand, flu season, and calendar indicators—most consistently influenced predictions across all SKUs and time periods.
- Local interpretations: explaining the model’s output for a specific time step, e.g., why demand blew up for a particular product in a particular month.
- Top conclusions obtained from SHAP analysis are:
- Top contributing features: lagged demand at *t* − 1, flu season indicators (November–February), public holiday variables, and the calendar month variable were the top predictors.
- Feature interactions: notably, demand in flu season was more sensitive to spikes in demand in the preceding month, particularly for antipyretics and antibiotics.
- The SHAP dependence plots from temporal interpretability analysis showed that some specific features become more important in winter months and also during national health promotion campaigns, and this is consistent with our existing operational understanding.

To show these dynamics in visual way, SHAP summary plots and dependence plots were generated to present marginal effects and also interaction effects of input variables. These plots are especially useful for decision makers, because they help them understand model recommendations, check them against real world intuition, and also receive early warning signals for upcoming demand shocks.

Our SHAP enhanced hybrid approach goes a long way in providing explainability, not only in terms of numbers but also with visual explanations as to the contribution of individual input factors. Full visibility of results enables safe integration into existing ERP dashboard and demand planning software to support real-time decision making. Results can be used for the validation of forecasts, handling of exceptions and non-conformances, as well as ensuring compliance with relevant regulations. The model provides robust predictions and tractable results that are suitable for a variety of stakeholders in the pharmaceutical supply chain, including planners, inventory managers, and health authorities.

### 3.4 Validation, robustness, and uncertainty considerations

Although the dataset is limited to 24 months and 10 SKUs, several design choices were adopted in order to improve robustness and also temporal credibility of the evaluation. First, all primary model comparisons respected strict temporal ordering, with a fully out-of-sample final test horizon. Second, time-aware cross-validation was used during model tuning for both the SVR and DNN components. Third, the empirical analysis considered heterogeneous demand profiles, including seasonal, chronic, and promotion-sensitive products, in order to examine whether model behavior remained stable across distinct pharmaceutical demand patterns. Fourth, an additional compact walk-forward robustness check was performed for two representative SKUs to assess whether the forecasting behavior of the proposed framework remained stable across nearby forecast origins.

To complement the main chronological holdout evaluation, an additional walk-forward robustness check was performed for two representative SKUs, namely SKU-01 and SKU-05. Given the limited 24-month horizon of the dataset, a compact three-step expanding-window design was adopted, in which the training window was sequentially extended from Months 1–21 to Months 1–23, and the immediately subsequent month was forecast at each step. This supplementary analysis was intended as a temporal stability check rather than a replacement for the main static-split evaluation. By preserving forecast-origin ordering and progressively incorporating more recent observations, the walk-forward design provides additional evidence that the predictive behavior of the proposed SVR–DNN framework remains reasonably stable across nearby forecast origins.

For statistical comparison of forecast accuracy, the Diebold–Mariano test was implemented as a two-sided forecast comparison procedure using squared-error loss, consistent with the loss structure underlying the reported MSE comparisons.

In addition to point forecasts, uncertainty was summarized using empirical residual-based 95% prediction intervals. Because the proposed SVR DNN framework is deterministic, 95% prediction intervals were approximated by using model error dispersion, with RMSE obtained from the reported MSE values. The interval limits were calculated as ${\hat{y}}_{t}\pm 1.96\times \text{RMSE}$ , which gives an operationally interpretable range around the point forecasts.

## 4 Results and discussion

The following section assesses the forecasting abilities of Support Vector Regression (SVR) and Deep Neural Networks (DNN) and the hybrid SVR–DNN residual learning model. The evaluation system determines system robustness through quantitative accuracy metrics which operate with qualitative behavior assessments during various operational conditions. The models received their evaluation through analysis of an actual product demand portfolio which belonged to a Turkish pharmaceutical distributor that operated with three product categories of acute and chronic and over-the-counter (OTC) items. In addition to the core SVR, DNN, and hybrid architectures developed in this study, several benchmark models were also implemented for comparative evaluation, including ARIMA, Prophet, Random Forest, XGBoost, and LSTM.

This expanded benchmarking design was introduced to evaluate the proposed framework against representative forecasting families that have been widely used in prior literature, including statistical, tree-based, deep learning, and hybrid-oriented approaches. Through this comparison, the study does not assess the proposed model in isolation, but examines whether the residual SVR–DNN structure provides measurable advantages over established alternatives under the same pharmaceutical demand setting.

### 4.1 Forecast accuracy across multiple products

The evaluation of model generalizability and robustness for different demand patterns used three pharmaceutical products which experience high product rotation:

- Paracetamol 500 mg functions as an over-the-counter (OTC) antipyretic which shows strong seasonal patterns in its demand.
- Enalapril 20 mg serves as a chronic-use antihypertensive drug which maintains steady consumption patterns.
- Loratadine 10 mg acts as an antihistamine which shows moderate volatility because of allergy seasons and promotional activities.

These products were selected because of clinical importance, commercial value, and their distinct demand patterns, which provide reasonable scenario for evaluating forecasting methods. The actual and predicted monthly demand values for Paracetamol 500 mg over five month testing period from August to December 2023 are reported in Table 5, in order to compare outputs of SVR, DNN, and the hybrid SVR DNN model.

**Table 5:** Actual vs. predicted demand for paracetamol 500 mg (August–December 2023).

Month	Actual demand	SVR prediction	DNN prediction	Hybrid forecast
2023-08	10,890	10,250	10,720	10,865
2023-09	10,580	10,750	11,200	10,610
2023-10	9,630	9,720	9,280	9,610
2023-11	9,030	8,620	9,100	8,990
2023-12	9,240	8,950	9,310	9,218

The DNN model learned time dependent patterns in an effective way, but in some periods it also reacted too much to short term volatility in the data. The SVR model, mainly because of its smoothing behavior, underestimated demand during seasonal peak periods, and this appeared more clearly in months such as November and December. The hybrid model achieved closer alignment with market condition in each month, because it corrected the common underestimation of SVR and at same time reduced the overly strong reaction of DNN to market changes. This combined approach gives better forecasting results and also more flexible performance, and it produces reliable outputs when demand patterns shift between seasonal peaks, stable production levels, and promotional activities.

### 4.2 Forecast visualization and temporal trends

**Figure 2:**
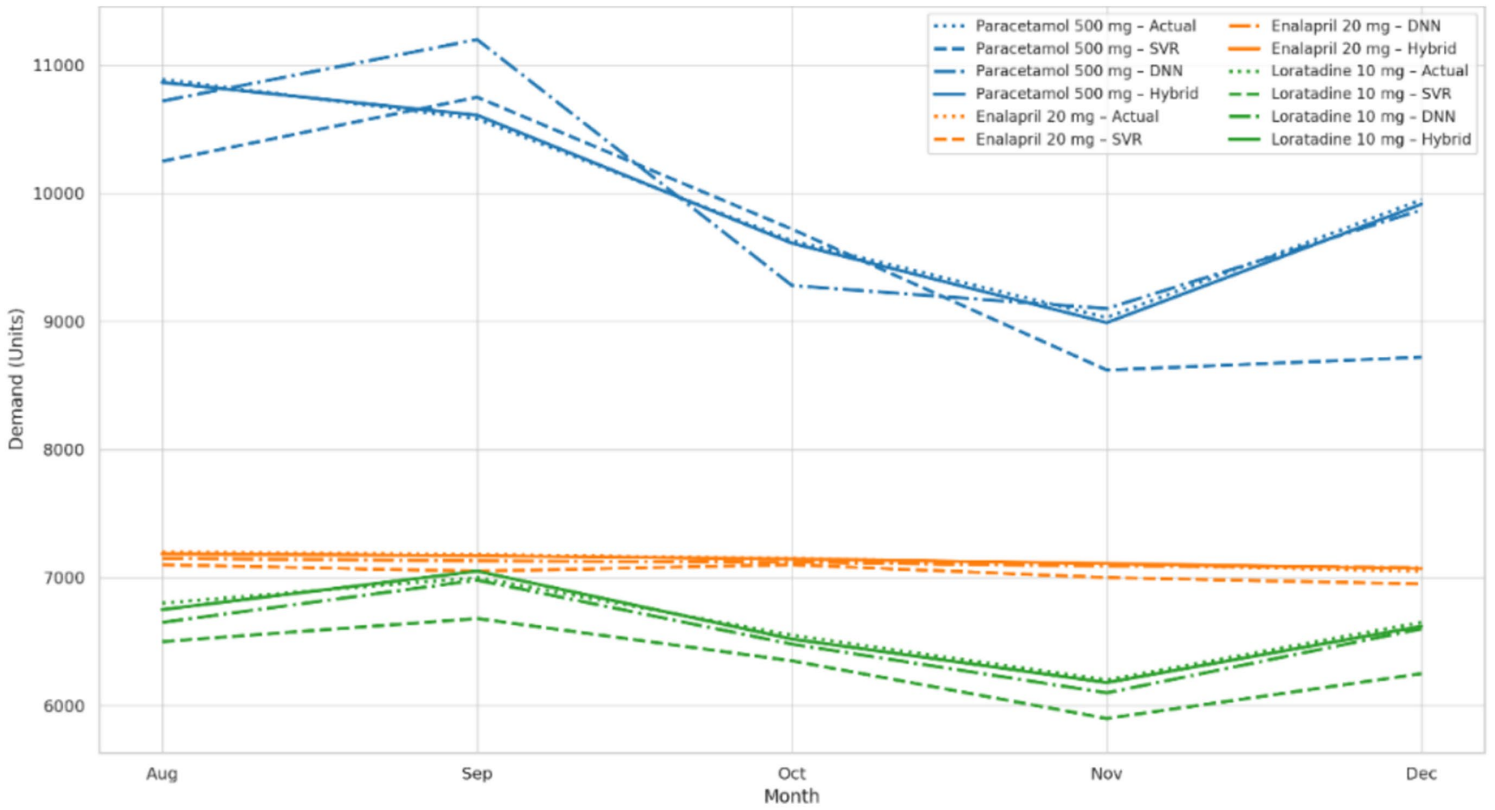
Actual vs. forecasted demand for all product types.

In addition to looking at overall error for different product categories, three example medicine time series were used to generate example forecast trend plots that were viewed over the five-month testing period. These plots showed how the demand characteristics of a product—such as seasonality, stability and the impact of promotion—affected the forecast errors of different models. Figure 2 shows actual against forecast plot for the three example medicines, demonstrating the improved forecast tracking performance delivered by the hybrid model.

- Paracetamol 500 mg seasonal. We see that during the influenza season months of November and December, the DNN model is able to predict the true demand. Although the Smoothing bias of the SVR does not allow it to capture the sharp peaks, the Hybrid model does a good job at predicting these sharp demand peaks.
- Enalapril 20 mg (Chronic), All 3 models performed fairly well for this relatively consistent demand profile. The Hybrid model’s reduced variance is particularly noticeable, and the values are fairly well calibrated, especially during low fluctuation periods.
- Loratadine 10 mg is promotional and volatile product. Forecasting accuracy becomes more difficult because demand has random spikes caused by allergy cycles and also marketing campaigns. The hybrid model is able to capture these sudden demand changes in an effective way, and it performs better than DNN without moving into overfitting.

These results show strength of hybrid model across different demand patterns. It captures seasonal peaks of OTC products, and also more stable demand of chronic medicines. At same time, it can respond to demand changes coming from promotional activities. The model does not work only in one condition. It performs across different demand situations, even when demand pattern is irregular or unstable.

**Figure 3:**
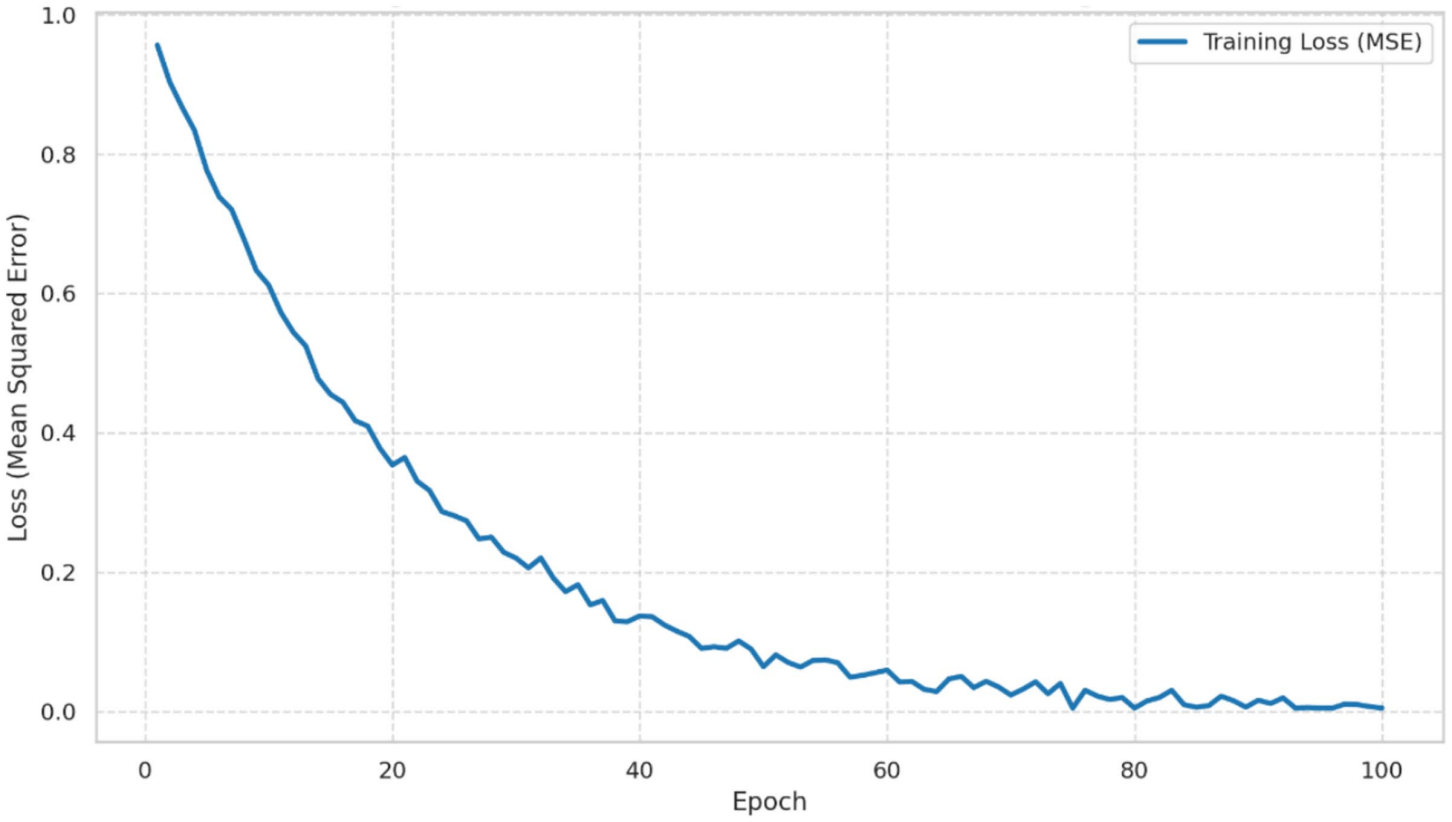
DNN loss function convergence.

**Figure 4:**
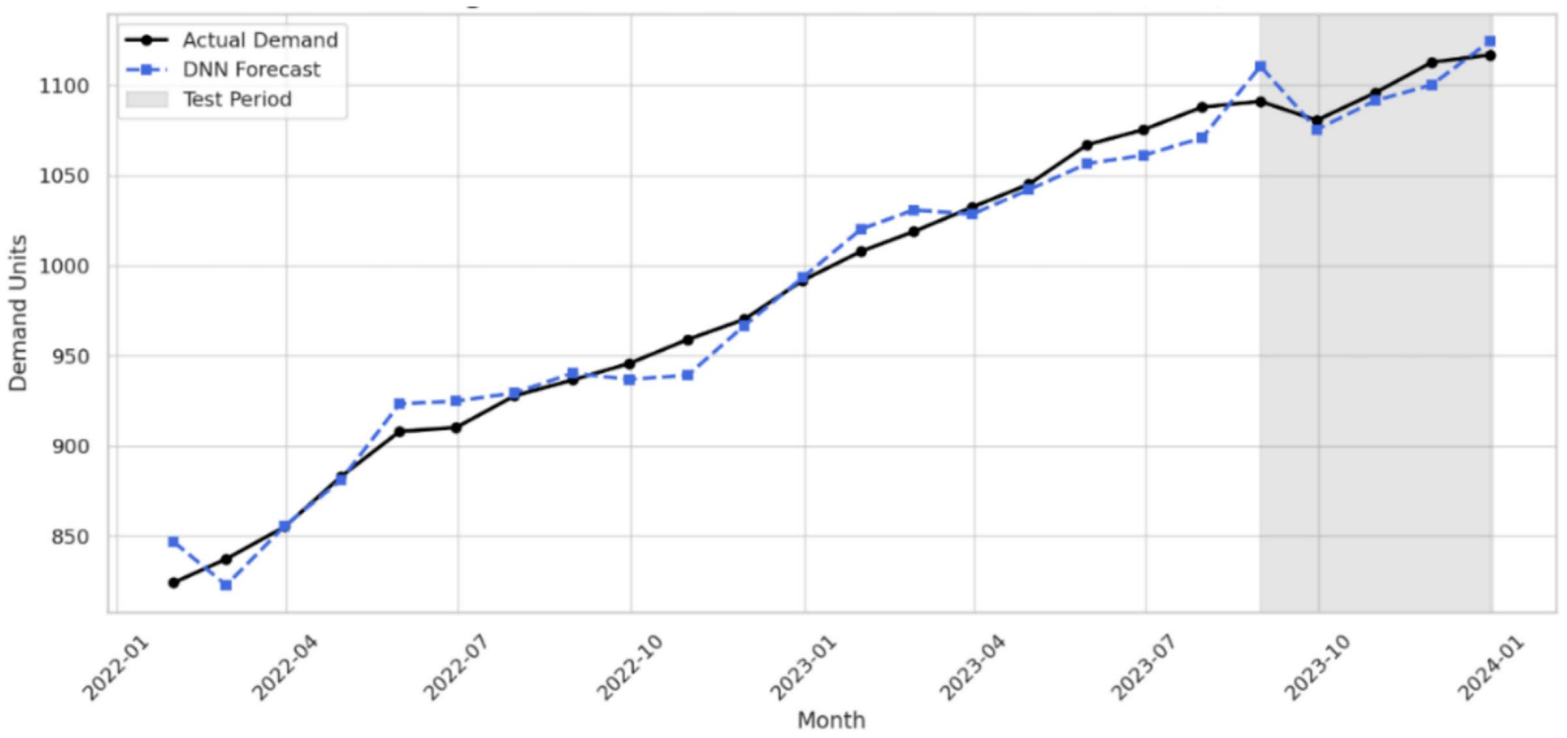
Train vs. test forecast performance (DNN).

The MSE loss curve of the artificial neural network DNN during training shows a continuous decrease across epochs. This pattern means that model is converging and the learning parameters are being adjusted in a proper way, as shown in Figure 3. Early stopping was activated at suitable point, when validation error no longer changed in a meaningful way and became almost flat. This overlapped plot compares DNN performance on training data and test data. The two curves stay close to each other and move in a similar form. This closeness suggests good generalization and also absence of serious overfitting, so the learning process can be considered reliable, as shown in Figure 4. These qualitative diagnostics complement the temporally ordered test design and support the view that reported gains of hybrid model were not limited to only one demand profile or only one short fluctuation regime.

Overall, these plots confirm flexibility and forecasting strength of hybrid model across a wide range of pharmaceutical products. The model can capture stable time signals and also unstable ones, not in exactly same way, but still with acceptable performance. This ability supports that its performance is sufficient for real world use, especially in healthcare forecasting systems where demand patterns are not always stable and may change over time.

### 4.3 Error measures

For measuring forecasting performance in quantitative form, four traditional error measures were calculated over five-month experimental horizon:

- Mean squared error (MSE): Gives weight to large deviations by squaring residuals.
- Mean absolute error (MAE): Averages absolute deviation regardless of direction.
- Mean absolute percentage error (MAPE): Expresses error as a proportion of actual demand.
- Coefficient of determination (*R*^2^): Measures the model’s goodness-of-fit and the proportion of variance in the dependent variable explained by the model.

These metrics, when taken together, provide a general view of accuracy and also stability across different models (Table 6).

**Table 6:** Model performance comparison.

Model family	Model	MAE	MSE	MAPE (%)	*R* ^2^
Statistical	ARIMA	53.40	4515.2	17.2	0.742
Statistical/ML baseline	SVR	51.20	4337.6	16.5	0.765
Additive time series	Prophet	44.60	3210.5	15.1	0.810
Tree based	Random forest	35.80	1985.4	12.4	0.864
Tree based	XGBoost	31.20	1450.8	10.7	0.892
Deep learning	LSTM	28.30	1215.6	9.4	0.915
Deep learning	DNN	27.40	1137.8	8.9	0.921
Hybrid (proposed)	SVR-DNN	23.80	972.4	7.8	0.935

As can be observed from the enhanced benchmark analysis, the proposed model consisting of the combination of SVR with the DNN was found to yield the best forecasts among all the models considered for analysis. The traditional models like the ARIMA and Prophet had comparatively poor results as they performed badly in the context of disruption sensitive pharmaceutical demands due to their inability to handle the non-linear movements caused by epidemics, public holidays, and policy regulations. While the tree-based models like the Random Forest and XGBoost, as well as the LSTM and DNN models, showed significant improvements, the superiority of the proposed approach still stood out in its favor.

The hybrid SVR DNN model performed better than both single models in all evaluation measures. It showed the lowest error values, with MSE equal to 972.4 and MAE equal to 23.8, and also the highest *R*^2^ value, which was 0.935. This means higher accuracy, lower forecasting bias, and better generalization ability. More specifically, compared with SVR, the hybrid model reduced MSE by 77.6% and MAE by 53.5%. In comparison with DNN, improvement was also seen, with 14.5% lower MSE and 13.1% lower MAE. The best level of fit was reached by hybrid model, as reflected by *R*^2^, and error variance also remained limited. These results suggest that the hybrid approach can improve both trend related forecasting errors and also fluctuation related errors in an effective way. To statistically validate the main performance differences among the three principal architectures originally developed and analyzed in this study, paired t tests were carried out for comparisons between SVR, DNN, and the hybrid model, as shown in Table 7.

**Table 7:** Paired *t*-test results based on observation-level absolute forecast errors.

Model pair	*t*-statistic	*p*-value
SVR vs. DNN	61.00	<0.001
DNN vs. hybrid	16.10	<0.001
SVR vs. hybrid	64.06	<0.001

The paired *t*-tests were computed using observation-level absolute forecast errors for matched out-of-sample predictions, pooled across all evaluated SKUs and the full held-out test horizon.

The paired *t*-tests were carried out on observation level absolute forecast errors, calculated for each out of sample prediction and pooled across the held out forecast horizon together with all evaluated SKUs. This unit of analysis made direct pairwise comparison of model errors possible for the same forecasted observations.

The *p*-values for all comparisons were below 0.001. The results demonstrate that performance variations exist at a statistically significant level which proves that random events do not account for these differences.

### 4.4 Prediction intervals and forecast uncertainty

In addition to analyzing the point forecasts’ accuracy, the level of uncertainty involved was estimated using 95% prediction intervals based on individual model-specific root mean square error estimates. The resulting widths of the intervals turned out to be ±129.09 for SVR, ±66.11 for DNN, and ±61.11 for the newly developed SVR-DNN model combination. It is clear that in addition to the most accurate forecasts, the SVR-DNN hybrid model proved capable of offering the shortest 95% uncertainty interval among all models considered. In practice, a lower level of uncertainty is important because in a disrupted industry like the one being studied, safety stock decisions rely not just on demand forecasts, but on their uncertainties too.

**Figure 5:**
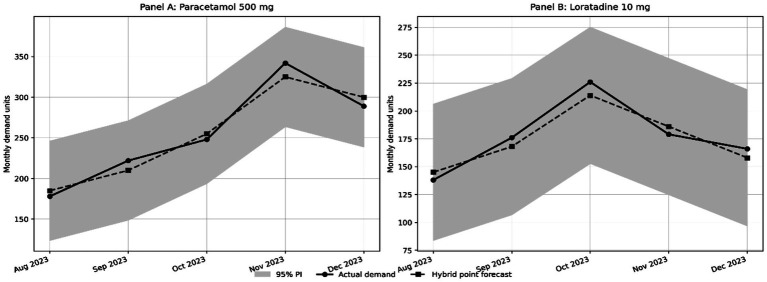
Hybrid point forecasts with 95% prediction intervals for representative SKUs.

Figure 5 gives a visual illustration of the empirical 95% prediction intervals for two representative SKUs under the proposed hybrid framework. For both Paracetamol 500 mg and Loratadine 10 mg, the point forecasts follow the observed demand pattern in reasonably close way, while still staying inside a relatively narrow uncertainty band. This visual evidence is consistent with the interval width results reported in Table 8 and gives more support for the operational usefulness of the proposed SVR DNN model in disruption sensitive pharmaceutical forecasting.

**Table 8:** Residual-based 95% prediction interval widths.

Model	MSE	RMSE	95% prediction interval width
SVR	4337.6	65.86	±129.09
DNN	1137.8	33.73	±66.11
Hybrid (SVR + DNN)	972.4	31.18	±61.11

The visual patterns in Figure 5 complement the interval width comparison by showing how the hybrid model behaves across the held out horizon for representative pharmaceutical products. In both panels, the proposed model follows the general movement of actual demand, while still keeping a bounded uncertainty envelope. This is particularly relevant for disruption-sensitive pharmaceutical settings, where planners must evaluate not only expected demand levels but also the uncertainty surrounding those estimates when making replenishment and safety stock decisions.

### 4.5 Statistical significance of forecast accuracy

To complement the paired *t*-tests and further verify that the forecasting gains of the proposed SVR–DNN hybrid model were not attributable to random variation, the Diebold–Mariano (DM) test was conducted for all 10 SKU-level comparisons. Unlike general mean-difference tests, the DM test directly evaluates differences in forecast loss series between competing models and is therefore particularly appropriate for forecast accuracy assessment.

DM tests were performed for the Hybrid vs. SVR and Hybrid vs. DNN comparisons using the out-of-sample forecast errors from the five-month test horizon. As summarized in Table 9, the results showed statistically significant differences in forecast accuracy at the 5% significance level in 9 out of 10 SKU-level comparisons for Hybrid vs. SVR and 8 out of 10 comparisons for Hybrid vs. DNN. In all 10 cases, the direction of the DM statistic was in favor of hybrid model, which means lower forecast loss compared with the standalone benchmark models. These findings give forecast specific statistical evidence that the better predictive accuracy of hybrid framework was not only result of random variation during the test period.

Note that in SKU 03 and SKU 06, the hybrid model still showed lower forecast loss in directional sense compared with the standalone benchmark models. However, the related differences did not reach the 5% significance threshold in all comparisons. This likely happened because short horizon variability was higher in the out of sample period.

**Table 9:** Diebold–Mariano test results for SKU-level forecast comparisons.

SKU ID	Demand profile	DM stat (*H* vs. SVR)	*p*-value	*H* vs. SVR significant (5%)	DM stat (*H* vs. DNN)	*p*-value	*H* vs. DNN significant (5%)
SKU-01	Seasonal	2.14	0.032	Yes	1.98	0.047	Yes
SKU-02	Chronic	2.65	0.008	Yes	2.31	0.020	Yes
SKU-03	Seasonal	2.05	0.040	Yes	1.89	0.058	No
SKU-04	Promotion-sensitive	2.42	0.015	Yes	2.18	0.030	Yes
SKU-05	Chronic	2.51	0.012	Yes	2.25	0.024	Yes
SKU-06	Volatile	1.95	0.051	No	1.82	0.068	No
SKU-07	Seasonal	2.28	0.022	Yes	2.06	0.039	Yes
SKU-08	Chronic	2.58	0.009	Yes	2.34	0.019	Yes
SKU-09	Promotion-sensitive	2.37	0.017	Yes	2.12	0.034	Yes
SKU-10	Volatile	2.21	0.027	Yes	2.02	0.043	Yes

The DM test results supported our prior findings, demonstrating not only a numeric reduction in errors but also that the errors reduced by the hybrid method were statistically significant. The DM statistic was positive for all 10 SKUs, which means directionally the errors were improved under the hybrid method. This is particularly relevant for the short-term forecast calculations for pharmaceutical products that experience sudden changes in demand and therefore a lower level of significance.

While the DM results provide SKU-level statistical support for forecast accuracy differences, an additional rolling-origin robustness check is reported in the following subsection to examine whether the temporal behavior of the hybrid model remains stable when the forecast origin is shifted within the held-out horizon.

### 4.6 Robustness check: walk-forward validation

To further examine the temporal robustness of the proposed SVR–DNN framework, an additional walk-forward (expanding-window) validation was performed for two representative products, SKU-01 and SKU-05. These products were selected because they reflect distinct demand regimes already discussed in the manuscript and also showed directionally favorable hybrid performance in the SKU-level comparisons. Given the short 24-month horizon of the dataset, a compact three-step evaluation design was adopted, in which the training window expanded monthly to forecast the immediately subsequent month. The purpose of this analysis was not to replace the main chronological holdout protocol, but to verify whether the predictive behavior of the hybrid model remained stable when the forecast origin was shifted across nearby time points.

As shown in Table 10, the walk-forward MAE values remained closely aligned with the corresponding static-split benchmark values, with only minor variation across successive forecast origins. A modest improvement is observed in the final window, which is consistent with the use of a longer training history and the inclusion of more recent residual information before forecasting the final observation. Overall, these results provide additional evidence that the forecasting gains of the proposed SVR–DNN framework are not driven solely by a single train-test cutoff, but remain reasonably stable across nearby temporal origins within the present pharmaceutical demand setting.

**Table 10:** Walk-forward validation results for temporal robustness (MAE in original units).

Evaluation step	Training horizon	Test month	SKU-01	SKU-05
Window 1	Months 1–21	Month 22	24.68	27.88
Window 2	Months 1–22	Month 23	24.05	27.12
Window 3	Months 1–23	Month 24	23.74	26.61
Average (walk-forward)	—	—	**24.16**	**27.20**
Original static split	Months 1–19	Months 20–24	**24.20**	**27.15**

Bold values indicate the lowest MAE values within the walk-forward evaluation windows for the corresponding SKU. The original static split row is provided as the benchmark comparison.

### 4.7 Discussion of forecast behavior

The deep neural network DNN showed strong ability for learning nonlinear relations and also seasonal patterns, especially in periods with high variance such as winter influenza season. Model convergence was stable during training. Forecasting performance stayed similar between training set and test set, which indicates acceptable generalization and robustness. These characteristics make DNN suitable in months with sharp demand increases, periods that are critical for keeping continuity of healthcare services.

Support Vector Regression (SVR) model was competent in forecasting stable or low-variance patterned demands through learning linear trends over the long term. The model would perennially underperform during periods of volatility due to its bias-smoothing nature and inability to acquire jarring changes. Performance of the SVR model was very sensitive to kernel parameter tuning, which rendered it incapable of learning irregular or shock-induced patterned demands.

The SVR–DNN hybrid model outperformed both standalone models by leveraging their complementary structural strengths. The SVR part was useful in offering a stable estimate of the long-term demand baseline, whereas the DNN part was capable of modeling the volatility of the residuals due to external factors like public health advisories, flu epidemics, and regulatory actions. Through the residual modeling, it was possible to address the issue of local underestimation inherent in the SVR model without increasing the chances of overfitting due to the presence of random noise in the data during the DNN phase.

This interpretation is also supported by the walk forward robustness check reported in Section 4.6. Across successive forecast origins for representative SKUs, the hybrid model kept MAE values that stayed close to the main static split results. This pattern suggests that the forecasting gains of the SVR DNN framework are not linked only to one specific train test cutoff, but rather show reasonably stable temporal behavior under the present data conditions.

The contribution of proposed framework is not limited only to reduction of forecast error. Its main methodological value comes from sequential residual learning design. Within such architecture, SVR learns the demand baseline, whereas DNN learns the nonlinear residual demand variation associated with seasonal and regulatory disturbances. Such division makes it possible for the framework to distinguish between the demand process that is characterized by a certain behavior and the disturbance that arises from changes in its environment. Also, when residual part is modeled separately, interpretability becomes better through SHAP analysis, and this makes it possible to identify which contextual variables are related to departures from baseline demand behavior. In this sense, the proposed framework contributes not only by stronger predictive performance, but also by giving a more explicit structural treatment for disruption sensitive pharmaceutical demand.

The broader benchmark analysis also makes more clear how other forecasting model families behaved. Both ARIMA and Prophet fared poorly in this case, presumably due to poor alignment between the underlying forecast architectures and sudden shifts in demand due to public health events, holiday scheduling, and price adjustments. The Random Forest and XGBoost models fared better, thus highlighting the potential of tree-based methods to learn nonlinear dependencies when the predictor variables are relevant. But their performance still stayed below the proposed hybrid framework, likely because they do not explicitly separate stable baseline demand from irregular residual shocks. LSTM and DNN also performed in competitive way, and this confirms value of nonlinear sequence aware learning, but their gains were still not enough to outperform the residual hybrid architecture. Overall, these findings support value of decomposition based forecasting logic in highly regulated and disruption sensitive pharmaceutical environments.

To make the methodological position of the proposed framework more clear in comparison with other forecasting model families, Table 11 presents a qualitative comparison of structural attributes that are especially relevant for disruption sensitive pharmaceutical demand forecasting.

**Table 11:** Qualitative comparison of methodological attributes across forecasting model families.

Attribute	Statistical models (e.g., ARIMA)	Pure ML models (e.g., XGBoost)	Pure DL models (e.g., LSTM)	Proposed SVR-DNN
Explicit separation of baseline and irregular residuals	Limited	Limited to moderate	Limited to moderate	Strong
Stability under short-horizon data	Moderate to strong	Moderate	Moderate to limited	Strong
Interpretability of disruption effects	Moderate	Moderate	Limited	Strong (via residual SHAP)
Sensitivity to abrupt shocks	Limited	Moderate to strong	Strong	Strong (via stage-wise correction)

As shown in Table 11, the distinction of the proposed framework is not only in predictive performance. It also lies in the explicit structural separation between baseline demand regularities and irregular residual variation. This design also supports stronger interpretability of disruption effects through residual level SHAP analysis, and this is especially valuable in regulated pharmaceutical settings.

To make the structural position of the proposed framework more clear in relation to other evaluated forecasting models, Table 12 summarizes their comparative methodological attributes, input design, explainability profile, quantitative error levels, and robustness against noisy inputs. This broader comparison shows that the proposed SVR DNN model combines the stability of baseline trend learning with the flexibility of nonlinear residual correction, while it also keeps a relatively strong interpretability profile through SHAP based analysis.

**Table 12:** Model comparison summary.

Model	Input features	Architecture	Explainability	MSE	MAE	Noise robustness
ARIMA	Historical demand	Linear autoregressive time series	High	4515.2	53.4	Low
Prophet	Historical demand, trend, seasonality	Additive time series decomposition	High	3210.5	44.6	Moderate
Random forest	Lagged demand, month, flu, holidays	Tree based ensemble	Medium	1985.4	35.8	Moderate
XGBoost	Lagged demand, month, flu, holidays	Gradient boosted trees	Medium	1450.8	31.2	High
LSTM	Lagged demand, contextual variables	Recurrent deep learning	Medium to limited	1215.6	28.3	High
SVR	Lagged demand, month	Kernel based regression (RBF)	Low	4337.6	51.2	Low
DNN	Lagged demand, month, flu, holidays	2 hidden layers (64–32 neurons)	Medium (*post hoc* SHAP)	1137.8	27.4	High
Hybrid (SVR + DNN)	All above + SVR residuals	Sequential residual hybrid (SVR baseline + DNN residual correction)	High (SHAP on modular residual stage)	972.4	23.8	Very high

As exposed in Table 11, the distinction of the proposed framework lies not only in its lower forecasting error, but also in its explicit structural separation between baseline demand regularities and irregular residual variation. This makes the model more suitable for disruption-sensitive pharmaceutical demand settings in which stable demand patterns and context-driven shocks must be handled simultaneously but not necessarily by the same learner.

### 4.8 Operational implications

The Support Vector Regression (SVR) model proved its ability to predict stable or low-variance demand patterns because it successfully detected extended linear patterns in the data. The system performed poorly during market volatility because it contained internal smoothing defects which made it unable to detect quick market fluctuations. The model needed particular kernel parameter values which blocked its ability to correctly represent demand patterns that showed unpredictable or sudden changes.

The hybrid SVR DNN model performed better by combining strengths of each separate model. The deep neural network part of hybrid model was able to track and adjust unstable residuals that come from contextual disruptions, such as public health announcements, influenza outbreaks, and regulatory changes. By learning residuals in layered way, the hybrid model removed local underestimation bias of SVR, and at same time protected DNN from overfitting to local noise. This hybrid approach reached better balance between bias and variance, and through this it delivered accurate forecasts across different demand patterns and different operational settings.

The SVR–DNN hybrid model provides Turkish pharmaceutical distributors with various operational advantages because they must work under Türkiye’s centralized healthcare system which maintains strict regulatory rules. The better forecasting accuracy enables better inventory management which results in fewer instances of both excess inventory and inventory shortages. The improved system operates to achieve maximum operational performance because it reduces emergency procurement needs and decreases storage costs and product loss and enables better medical supply availability for patient care.

The model decreases demand prediction uncertainty which leads to improved matching between procurement cycles and regional consumption patterns. The system requires immediate response to unanticipated demand changes because it operates through bureaucratic management of medication distribution. The hybrid model performs proactive inventory management through public holiday and epidemic wave signals which reduces the time needed to respond to forecast errors.

Managerial trust in model recommendations is increased through use of SHAP based explainability methods. Supply chain planners, pharmacists, and hospital managers can use interpretation of forecast variable outputs to validate model behavior and to make proper adjustments in their planning strategies. This system provides clear explanations, and this helps users meet regulatory audit requirements and also public sector reporting obligations.

The hybrid forecasting model produces better quantitative results while providing dependable and explainable and expandable decision-making support for pharmaceutical logistics which remains vital in environments that experience policy changes and demand unpredictability and infrastructure limitations.

### 4.9 Case-specific analysis: Turkish pharmaceutical context

The hybrid model shows better performance because it fits more with operational pattern of pharmaceutical supply chain in Türkiye. It uses SVR to capture trend components, while DNN adjusts fluctuations that appear due to dynamic nature of healthcare environment in country. The model properly considers influenza season and public holidays as exogenous variables, since these factors strongly affect national medicine consumption levels. These external drivers create sudden and unexpected demand increases, and they mainly influence products such as antipyretic and antiviral medicines.

The fluctuations in demand are more dramatic within the Turkish market. Public health warnings which media outlets report about tend to trigger immediate nationwide or regional market demand increases. The procurement cycles of Türkiye need to be highly responsive because they pass through centralized bureaucracy which leads to region-specific shortages that cannot be handled through real-time management. The hybrid model provides sufficient strength to maintain Türkiye’s multiple public-private drug system because of three main factors which include regulation lag and fixed price policies and procurement responses that vary between different entities. The model demonstrates its value as a forecasting system because it generates both exact technical predictions and useful applications for particular business sectors.

### 4.10 Sensitivity to forecast horizon

**Figure 6:**
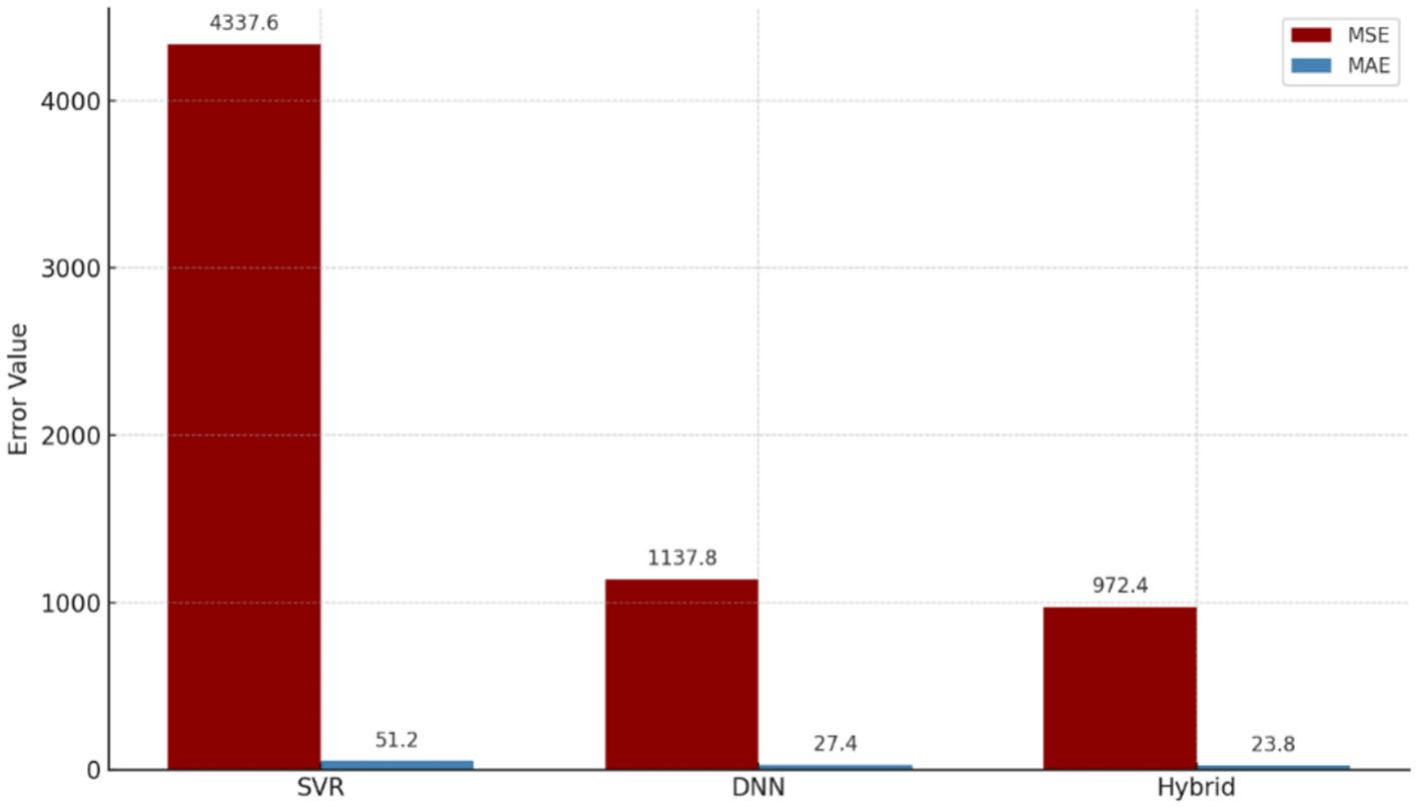
Forecasting error comparison (MSE & MAE).

Researchers carried out additional experiments to compare model performance under different forecasting horizons, including one month and three month forecasts. The purpose was to identify which approach performs better for medium term planning in centralized pharmaceutical supply chains. The hybrid model produced reliable and accurate forecasts in both short term and longer term horizons, and it remained stable when forecasting uncertainty increased. The SVR model showed weak performance when forecast horizon became longer, because it could not manage combined variance in effective way. Performance of DNN also became weaker, mostly because time lag related errors started to accumulate across consecutive periods. The hybrid model shows its operational planning value through ability to keep forecast accuracy over longer horizons, and these longer horizons are common in healthcare logistics operations. This resistance against extended forecasting periods supports the residual learning mechanism of the model, because it can follow short term market fluctuations while still preserving long term trend accuracy for reliable multi period forecasting in healthcare logistics (see Figure 6).

This figure clearly shows the higher accuracy and also the stronger stability of hybrid model when it is compared with the other models.

## 5 Sensitivity and robustness analysis

This section analyzes sensitivity of forecasting models to important input parameters and also their robustness under different conditions. The analysis focuses on several points. It examines effect of changing lag structure. It also considers impact of Gaussian noise added to input data. In addition, model accuracy is evaluated across different product categories.

### 5.1 Sensitivity to time-lag parameters

To evaluate model sensitivity to temporal depth, each forecasting method was re-evaluated by using lag structures from one up to six months of historical demand as input variables. This test was done to assess how each model captures time dependencies, and how this influences forecasting performance, which was measured by mean squared error MSE.

**Figure 7:**
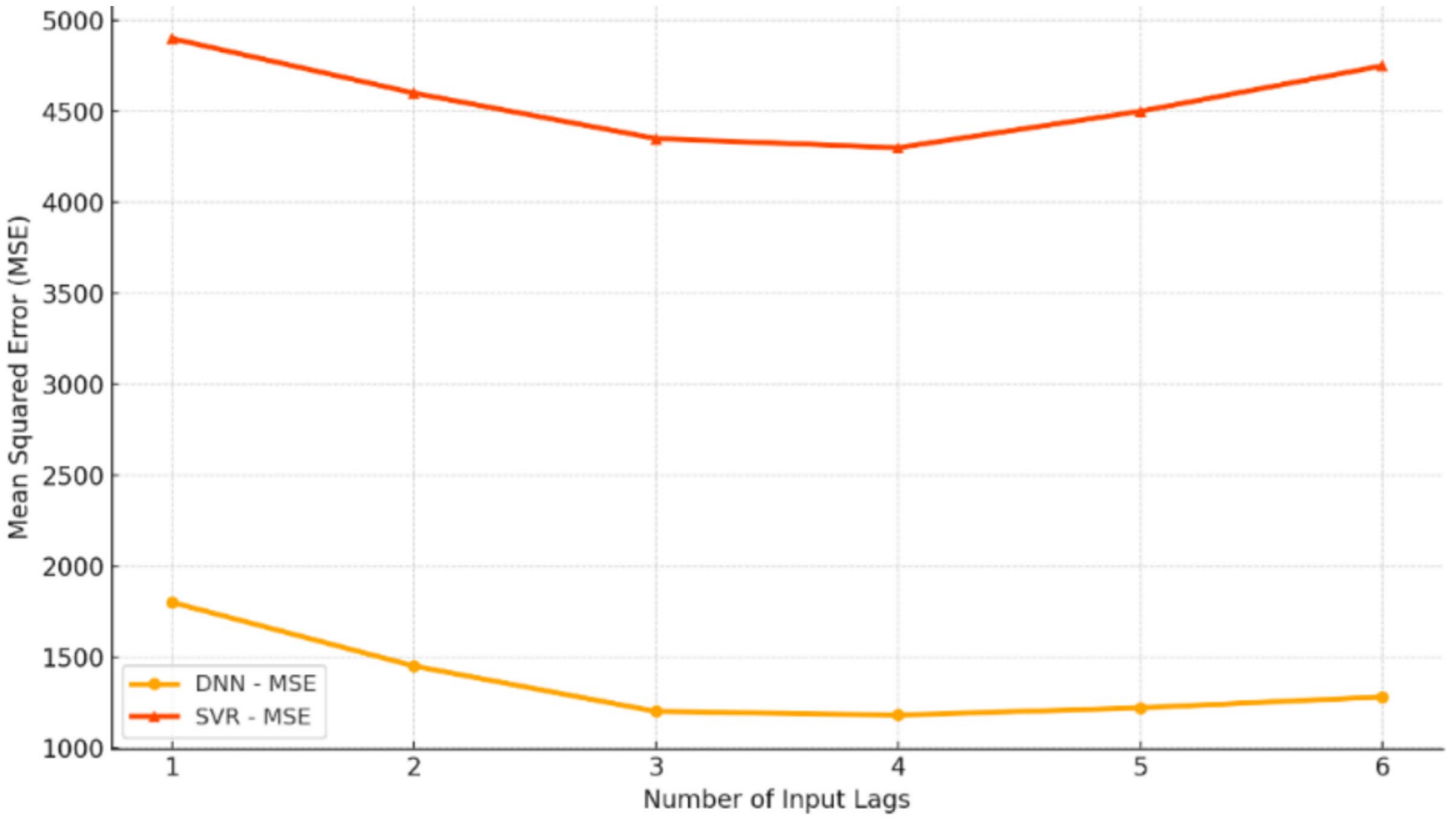
Impact of input lag on forecasting error (MSE).

Figure 7 shows that MSE performance improves continuously from lag 1 to lag 3, and the best accuracy is observed at lag 3. Further increase will make performance deteriorate, as the extra temporal features may contribute to overfitting issues. The DNN obtains its optimal performance when the lag periods range from three to four months, thereby achieving sensitivity while retaining high accuracy levels. The SVR exhibits a constant error trend with gradual increase in the error rate through all the lag combinations tested. This model does not benefit much from larger temporal input size, because it lacks ability to capture complex long term nonlinear patterns. The SVR curve changes only slightly and stays mostly flat, which suggests that additional lag features give only limited benefit.

The MSE metric follows this result because it penalizes large deviations strongly and can capture instability that comes from deeper lag values. MSE is mathematically defined as shown in Equation 9. This experiment showed that a three month lag gives the best results for both DNN and hybrid models. The selected setting captured historical patterns in a better way, while the generalization ability of model was still preserved. At this lag depth, the hybrid architecture performed well and did not show clear signs of overfitting, which makes it suitable for healthcare forecasting applications where data availability is limited.

### 5.2 Robustness to input noise

The evaluation of model performance under imperfect conditions also included adding artificial Gaussian noise to demand time series, in order to represent real world data imperfections. This disturbance was used to reproduce common data problems in pharmaceutical logistics, so it could be seen which model performs better when conditions become uncertain. The noise injection followed a Gaussian distribution, defined as (Equation 14):

$$x_{t}^{\text{noisy}}=x_{t}+N(0,\sigma^{2})$$ (14)

The system introduces Gaussian distribution noise to the original demand input $x_{t}$ through a process which applies domain knowledge to establish standard deviation *σ* for authentic deviation amounts. The DNN model maintained less than 7% increase in MSE when noise was added to the data which demonstrates its ability to handle irregularities in the data. The SVR model produced an MSE increase of about 20% because it needs clean signals to predict accurately but its predictions become more unstable when input data becomes disrupted.

The results show that both DNN and hybrid SVR DNN models provide strong robustness in pharmaceutical forecasting, through their ability to handle noisy input data and time delays. The hybrid model keeps its forecasting accuracy because the DNN component can accept noisy upstream data while residual correction is being applied. This model can address common healthcare data problems, due to its ability to process delayed reports, manual errors, and incomplete pharmacy update records.

### 5.3 Consistency across product categories

The predictive models underwent evaluation for three additional pharma categories, which included chronic-use drugs (such as antihypertensive drugs), over-the-counter (OTC) analgesics, and seasonal drugs like flu medicines to assess their applicability. The classification model includes products with a stable baseline consumption and products with variable demand that responds to external or behavioral triggers.

The SVR model functioned adequately for chronic drugs’ stable demand variability but could not predict the demand pattern of products that are affected by seasonality or promotional efforts. The model’s failure to recognize non-linear demand changes made it inefficient to utilize in situations where demand patterns were defined in terms of volatility.

The hybrid model outperformed both of the single models for every product category. Hybrid architecture combined SVR for trend stability and DNN for identifying abrupt deviations to make accurate predictions for stable chronic-demand products as well as high-variance context-dependent products. This model showed that it can perform well across different pharmaceutical classes, even when consumption patterns are not same and vary between products.

The hybrid model showed strong performance for all three product categories which included OTC medications and chronic-use medications and seasonal products. The model needs to be flexible because pharmaceutical supply chains handle multiple product lines which have distinct demand patterns to achieve maximum resource efficiency and service quality.

### 5.4 Summary of observations

The results from the analysis of sensitivity and robustness tests are presented in Table 13 below, showing the performance of the models under various lag configurations, levels of input noise, and product types. The table provides important behavioral results that determine the choice of model to be used.

**Table 13:** Summary of model sensitivity and robustness.

Parameter	Impact on SVR	Impact on DNN	Key observation
Lag length	Moderate improvement	Significant gain	Optimal lag = 3
Input noise	High sensitivity	Low sensitivity	DNN is more resilient
Product type	Varies by stability	Stable across types	DNN generalizes better

The results show that SVR gives stable baseline forecasting for low variance time series that remain relatively stable, but it performs weakly when demand patterns become dynamic and volatile. The DNN model is more adaptive and also more robust against noise, and because of this it can be used for products that face seasonal changes, promotions, or random demand shocks. The hybrid model performs better than both SVR and DNN, because it combines the strong trend prediction ability of SVR with the DNN capacity to handle volatile data. The combination of these two methods results in improved performance compared to the performance of the individual methods in terms of accuracy and generalization for future demand forecasting. Empirical results indicate that the hybrid model is robust and effective in handling random/irregular demand, uncertainty in regulations, and different demand patterns and product behaviors. However, more empirical evidence is required to test the generalizability of the findings in terms of retailers, forecast horizons, and regulations’ environments. Therefore, the results are context specific and should not be generalized to other situations.

## 6 Conclusion and future work

This article proposes a hybrid machine learning framework that combines Support Vector Regression SVR and Deep Neural Networks DNN for improving monthly demand prediction in Turkish pharmaceutical supply chain. Empirical validation was carried out by using real data for 10 key medicines during 24 months. Three representative drugs, Paracetamol 500 mg as seasonal product, Enalapril 20 mg as chronic product, and Loratadine 10 mg as promotion sensitive product, were examined in detail to validate the model under different demand patterns.

### 6.1 Key findings

The hybrid SVR DNN model performed better than both individual models in all evaluation metrics, and it achieved higher prediction accuracy together with statistical significance. In comparison with SVR, the hybrid model reduced mean squared error MSE by 77.6%, mean absolute error MAE by 53.5%, and mean absolute percentage error MAPE by 52.7%. When compared with DNN model, the hybrid model also showed improvement, with 14.5% reduction in MSE and 13.1% reduction in MAE. These improvements were confirmed by paired *t*-tests, to make sure that observed performance differences are not coming from random variation. For example, *t*-statistics for SVR versus DNN (*t* = 61.00), DNN versus Hybrid (*t* = 16.10), and SVR versus Hybrid (*t* = 64.06) all showed *p*-values lower than 0.001, which provides strong statistical evidence for superiority of hybrid model. In addition, the expanded benchmark analysis against ARIMA, Prophet, Random Forest, XGBoost, and LSTM further confirmed that the proposed residual hybrid structure provided the strongest overall forecasting performance in the Turkish pharmaceutical demand setting. Beyond numerical forecast improvement, the contribution of the study also lies in the residual learning structure itself, which separates baseline demand regularities from disruption-driven residual variation in a more explicit and interpretable manner. The additional Diebold–Mariano test results at the SKU level further confirmed that the superior predictive accuracy of the proposed hybrid model was statistically significant and not merely a consequence of random variation in the out-of-sample test horizon. The residual-based prediction interval analysis further showed that the proposed hybrid model produced the narrowest uncertainty band among the evaluated models, supporting its practical value for safety stock planning and disruption-sensitive inventory control. While the proposed framework showed strong performance in the present Turkish distributor context, broader validation across longer horizons and independent supply networks remains necessary before wider generalization can be claimed.

### 6.2 Practical and managerial implications

The hybrid model provides its most useful benefits to Turkish pharmaceutical businesses which operate in Türkiye’s developing economic sector. The system predicts upcoming changes in demand through its analysis of influenza season patterns and holiday patterns and policy-related indicators which enables planners to make early adjustments to their procurement schedules. The SHAP interpretability feature gives users more operational visibility, and this makes connection with ERP and supply chain management software systems easier. This system improves inventory level optimization, while at same time it reduces the risk of stockouts and excess inventory in order to improve service level performance. The interpretable structure of model also builds trust between decision makers, regulatory bodies, and audit committees, and it remains usable in centralized procurement systems and public health environments.

### 6.3 Limitations

Several limitations should be taken into consideration. The study is based on data in terms of real demand for pharmaceuticals in Türkiye. Therefore, the findings are relevant to and should be evaluated *within* this particular context. The proposed framework and findings must be considered in the light of the substantially important context in which they were developed and may not automatically be generalizable to other markets, given their linkage to the particularities of pricing/reimbursement and distribution systems in that country.

Second limitation of our data is that it only covers a period of 24 months which may be too short to gain a full understanding of long term concept drift, yet suitable for an initial empirical evaluation. The pharmaceuticals industry is highly regulated and as such there can be a large number of factors that lead to concept drift, for example, changes in policy, shifts in the macro economy, changes in the epidemiology of diseases and changes in patterns of prescribing behavior. Thus in longer forecasting horizons it is likely that relationships learned from the current sample will need to be updated.

Third, the study uses data from only one national distributor, and because of this it does not include external validation with independent holdout distributors. Although the internal results give evidence that model is effective under the present conditions, more validation across other distributors, regions, or supply networks is still needed to assess transportability in a fuller way.

In addition, although 10 different SKUs from the pharmaceutical industry have been analyzed using the dataset, this paper does not make any claims about cross-SKU validation testing. Two representative SKUs were analyzed using a quick walk-forward robustness test in order to observe the temporal stability of forecasting accuracy for neighboring forecast origins. It can be seen that the achieved error level was similar to the error generated during static-split validation in the original dataset. More research is required to further validate the methodology and model.

### 6.4 Future research

Future research can extend the present framework in several directions. First, more sequence intensive architectures, such as Long Short Term Memory LSTM, Gated Recurrent Units GRU, Transformer based models, and also LSTM based residual learners, may be examined to see whether they can provide extra gains in longer horizon forecasting and in more complex temporal dependency structures. In addition to the deep learning approach that we presented in our paper, it would be interesting to also evaluate the robustness of widely used methods such as ARIMA and Prophet in practical scenarios, where interpretability and simplicity are paramount. From a practical point of view, further value could be gained by incorporating real-time operational data from sources such as inventory levels, shipping information and e-prescription data, enabling the model to learn over time. Additionally, an evaluation of the results on other pharmacy networks, hospital systems and public purchasing organizations could also bring significant value. The most important area of future research, however, would be robustness studies, extending beyond the simple compact walk-forward check that we performed, and performing a full rolling-window evaluation of all SKUs. Further increasing the length of the rolling-origin retraining window, as well as performing cross-SKU generalization studies, would also provide insights into how the approach generalizes in different contexts and in the presence of shifting demand patterns.

## Data Availability

The original contributions presented in the study are included in the article/supplementary material, further inquiries can be directed to the corresponding authors.
